# Relationship between oxidative stress and COVID-19 severity: A systematic review and meta-analysis

**DOI:** 10.5455/javar.2025.l957

**Published:** 2025-09-22

**Authors:** Mohammad Shah Alam, Mohammad Nazmol Hasan, Md. Arman Sharif, Md. Shahidul Islam, Md. Mizanur Rahman, Rafee Shahrier, A. B. M. Rubayet Bostami, Mousumi Saha, Md. Sodrul Islam, K. H. M. Nazmul Hussain Nazir

**Affiliations:** 1 Department of Anatomy and Histology, Gazipur Agricultural University, Gazipur, Bangladesh; 2 Department of Agricultural and Applied Statistics, Gazipur Agricultural University, Gazipur, Bangladesh; 3 Department of Physiology and Pharmacology, Gazipur Agricultural University, Gazipur, Bangladesh; 4 Department of Agricultural and Applied Statistics, Bangladesh Agricultural University, Gazipur, Bangladesh; 5 Department of Animal Science and Nutrition, Gazipur Agricultural University, Gazipur, Bangladesh; 6 Department of Surgery and Radiology, Gazipur Agricultural University, Gazipur, Bangladesh; 7 Department of Microbiology and Hygiene, Bangladesh Agricultural University, Mymensingh, Bangladesh

**Keywords:** COVID-19, SARS-CoV-2, oxidative stress, antioxidants, meta-analysis

## Abstract

**Objective:**

COVID-19 is a complex disease in which the interaction of the SARS-CoV-2 virus with target cells, activation of the immune system, and release of inflammatory cytokines are closely intertwined. Oxidative stress is associated with all of these events, which significantly contribute to the pathogenesis of COVID-19. This study aimed to analyze the relationship quantitatively between oxidative stress and the disease severity in hospitalized patients.

**Methodology:**

Articles measuring pro-oxidant and antioxidant markers in patients with COVID-19 were retrieved through the search engines ScienceDirect, PubMed, and Google Scholar. Two authors independently extracted data using the data extraction tool, and a third arbitrator was consulted if consensus was not reached. Data were subjected to meta-analysis using the “meta” package of R programming for forests and the trim and fill method under a random-effects model based on standardized mean differences (SMDs). We tested for heterogeneity in effect size using the *I^2^* statistic and Egger’s test to assess bias.

**Results:**

Of the 3,103 articles screened, 17 met the inclusion criteria. When comparing control *vs.* mild cases, control versus severe cases, and mild versus severe cases, hydrogen peroxide (H₂O₂) levels were significantly increased [(SMD, 2.46; CI: –0.81 to 5.73; *p* = 0.05), (SMD, 3.22; CI: –0.70 to 7.14; *p* = 0.05), and (SMD, 0.49; CI: –0.23 to 1.20; *p <* 0.05), respectively]. Similarly, total oxidative stress (TOS) levels were significantly increased when comparing control versus mild cases (SMD, 4.01; CI: 0.85 to 7.18; *p* = 0.01), control versus severe cases (SMD, 6.51; CI: –0.59 to 13.62; *p* = 0.07), and mild versus severe cases (SMD 3.07; CI: 01.21 to 7.36; *p* *=* 0.05). However, superoxide dismutase (SOD) levels were decreased when comparing control versus mild cases (SMD, –0.60; CI: –1.31 to 0.12; *p* = 0.05), control versus severe cases (SMD, –1.68; CI: –4.00 to 0.64; *p* = 0.05), and mild versus severe cases (SMD, –0.73; CI: –1.81 to 0.36; *p* = 0.06). Similarly, catalase and glutathione levels were decreased when comparing control versus mild cases, control versus severe cases, and mild versus severe cases. Moreover, thiol levels were significantly decreased when comparing control versus mild cases (SMD, –1.72; CI: –2.91 to –0.53; *p* = 0.005), control versus severe cases (SMD, –2.83; CI: –3.97 to –1.69; *p* = 0.00), and mild versus severe cases (SMD, –1.19; CI: –1.83 to –0.54; *p* = 0.00).

**Conclusion:**

This meta–analysis revealed significantly higher levels of pro-oxidants (H₂O₂ and TOS) and lower levels of antioxidants (SOD, CAT, GSH, and thiols) in severe cases of COVID-19 compared to controls and mild cases, indicating that oxidative stress contributes to the severity of the disease. Assessing pro-oxidant and antioxidant stress markers may help assess disease severity for effective triage of COVID-19 patients. This information will be valuable for a broader discussion on the pathogenesis of COVID-19.

## Introduction

Coronavirus disease 2019 (COVID-19), caused by the severe acute respiratory syndrome coronavirus-2 (SARS-CoV-2), is the largest coronavirus pandemic in history [1]. As of June 26, 2025, the disease has been reported worldwide with 778 million infections and more than 7.0 million deaths. The SARS-CoV-2 virus infects humans by binding to an angiotensin-converting enzyme 2 (ACE-2) on the mucosal surface of the respiratory tract [2]. The virus initially replicates in the respiratory system’s epithelial cells [3, 4]. The virus can also infect epithelial cells lining the digestive, cardiovascular, and central nervous systems, as high expression of ACE-2 has been reported in these systems [4, 5].

Clinically, the disease is mainly characterized by mild, moderate, and severe conditions. While most patients with COVID-19 (approximately 80%) experience asymptomatic to mild symptoms such as cough, fever, and fatigue, 15%–20% of patients develop moderate to severe symptoms of pneumonia, and 3%–5% of patients become severely ill with conditions such as acute respiratory distress syndrome, acute shock, and/or multiple organ failure [6, 7]. Pathologically, severe/critically ill COVID-19 is characterized by “cytokine storm or cytokine release syndrome,” a hyperinflammatory response associated with excessive secretion of pro-inflammatory cytokines and an overreaction of the immune system [6, 8, 9]. Cytokine storm, a poor prognosis of the disease, is associated with the worst outcomes and highest mortality rates in COVID-19 patients [10–12]. Epidemiological studies suggest that COVID-19 severity correlates with underlying comorbidities, including diabetes, hypertension, chronic respiratory and kidney diseases, cancer, obesity, cardiovascular disease, immunosuppressive conditions, and aging [13]. Elevated levels of reactive oxygen species (ROS) have been observed in patients with underlying comorbidities [14]. Interestingly, elevated levels of ROS have also been linked to the pathophysiology of COVID-19, including endothelial cell dysfunction, blood clotting, microvascular thrombosis, and platelet aggregation, which ultimately contribute to the severity and mortality of COVID-19 [12]. Therefore, oxidative stress and associated inflammation are now recognized as important contributors to COVID-19 pathogenesis and severity [15].

When the body accumulates excess ROS and overwhelms the antioxidant defenses of cells and the body’s ability to detoxify toxic effects, it induces oxidative stress [16–18]. The most damaging ROS to cells includes superoxide anion (O₂•−), hydroxyl anion (•OH), and hydrogen peroxide (H₂O₂), while intracellular antioxidants in the human body include superoxide dismutase (SOD), glutathione (GSH), and catalase (CAT). SOD catalyzes the dissociation of O₂•− to H₂O₂, which, in turn, is decomposed to H₂O and O₂•− by CAT [19]. Moreover, H₂O₂ is converted to H₂O by glutathione peroxidase (GPx), which is recharged by glutathione reductase (GSR), which is itself reactivated by glutathione (GSH) [20, 21]. Optimal levels of GSH are essential for the activity of the GPx and GSR systems, where GSH serves to restore each enzyme to its active state [22], thereby maintaining pro-oxidant and antioxidant homeostasis in the body. Furthermore, thiols play an important role in cellular antioxidant defense and redox signaling [23, 24].

Our recent meta-analysis on a potent antioxidant supplement, N-acetylcysteine (NAC), showed improved clinical outcomes of COVID-19, specifically, increased oxygen saturation, significant reductions in inflammatory marker levels, and reduced mortality [25]. Several narrative reviews have reported a strong link between COVID-19 pathogenesis and oxidative stress [14, 26, 27]. Furthermore, several observational and cohort studies have reported significant changes in ROS and antioxidant levels in severe COVID-19 patients [28–33]. However, there are no studies that quantitatively analyze the relationship between oxidative stress and the severity of COVID-19. Therefore, we conducted a meta-analysis of pro-oxidant and antioxidant markers in hospitalized COVID-19 patients and healthy individuals.

## Materials and Methods

### Ethical approval

Meta-analysis was exempt from ethical approval because we collected or synthesized data from previously published research articles in which the corresponding authors noted ethical approval.

### Search strategy

We identified studies that published data on pro-oxidant and antioxidant markers in confirmed COVID-19 patients. We used the PRISMA guidelines to search electronic databases (S1; PRISMA checklist). We retrieved studies from PubMed, Google Scholar, and ScienceDirect. The search included medical subject headings (MeSHs) terms, keywords, combinations, and snowball searches to retrieve relevant papers. We used search terms independently and/or together using various Boolean operators such as “OR” or “AND.” The keywords and phrases were “COVID-19”, “SARS-CoV-2”, “novel Coronavirus”, “oxidative stress”, “redox imbalance”, “antioxidants”, and “enzymatic antioxidants”. Using those keywords, the following search map was applied: (blood OR complications OR diagnosis OR immunology OR mortality OR pathology OR physiopathology) AND COVID-19 [MeSH Terms] AND (adverse effects OR genetics OR immunology) AND oxidative stress [MeSH Terms] AND (adverse effects OR metabolism OR pharmacology OR therapeutic use) AND antioxidants [MeSH Terms] on the PubMed database (S2). Thus, the PubMed search combines #1 AND #2 AND #3. Full-length articles published in English on confirmed COVID-19 patients aged 19 to 80+ years. Four researchers independently assessed the search results. The articles searched were published from January 2020 to April 2025.

### Study selection/eligibility criteria

Retrieved studies were exported to EndNote version 21 to remove duplicate studies. We screened the selected studies using their titles and abstracts before retrieving the full-text articles. We followed pre-specified inclusion and exclusion criteria to screen the full-text articles. Discrepancies were considered for the final selection of studies for inclusion in this study.

### Inclusion criteria

Articles were included in this meta-analysis if they met the following criteria: (1) studies that evaluated pro-oxidant and antioxidant markers in patients with COVID-19; (2) data on oxidant and antioxidant parameters in mild, severe, dead/surviving COVID-19 patients; (3) randomized control trials, cross-sectional, observational, case-control, prospective, and cohort studies measuring pro-oxidant and antioxidant markers in serum, plasma, or other tissues of patients with COVID-19; (4) the disease (COVID-19) was diagnosed according to the standard protocol recommended by the World Health Organization, where multiple tests were performed on the same sample, including PCR, antigen rapid diagnostic methods, biochemical tests, radiological, clinical history, and signs and symptoms; (5) human subjects; (6) English language; and (7) studies that provide case numbers, means, and standard errors/deviations.

### Exclusion criteria

Articles were excluded from this meta-analysis if they met the following criteria: (1) conference summaries, correspondence, editorials, meta-analysis, and review papers; (2) animal studies; (3) dual publications (if the same data were used in multiple publications, the article that provided the strongest evidence was considered for this analysis); (4) non-English articles; and (5) insufficient data.

### Quality assessment

The quality of the studies was assessed based on the Newcastle–Ottawa Scale (NOS) [34]. A total NOS score ≥ 7 indicates a good quality of the included studies. The NOS score is presented in Table 1.

### Participant

Participants were divided into three groups: control, mild, and severe COVID-19. COVID-19 was diagnosed by multiple methods on the same sample, including PCR, rapid tests, biochemical, clinical, and radiological/CT scan parameters. The “control” group consisted of healthy participants. The “mild” group included confirmed COVID-19 cases with cough, fever, and fatigue, but no typical pneumonia changes on CT scan/radiology. The “severe” group included confirmed COVID-19 cases with severe pneumonia, organ failure, and respiratory distress.

### Meta-analysis

We developed a data extraction tool in an Excel sheet, and the following data from eligible studies were extracted: author, study location, sample size, number of mild and severe patients, pro-oxidant and antioxidant markers, study type, and publication NOS score. This study used oxidant markers, including H₂O₂, O₂•−, and TOS, and antioxidant markers, including SOD, CAT, GSH, NO, and thiols. Two authors (Shah Alam and Hasan) independently extracted data using the data extraction tool, and a third arbitrated if consensus was not met. Data were subjected to meta-analysis using the “meta” package in R for forest plots under a random-effects model based on standardized mean difference (SMD), and adjusted SMDs from the trim and fill approach were also used to predict COVID-19 severity from control to mild, control to severe, and mild to severe [35]. However, adjusted SMD could be computed for more than two studies [36, 37]. The *I^2^* statistic was used to calculate the heterogeneity between the studies, and a *p*-value of less than 0.05 indicated significant study heterogeneity. If the value of *I^2^* is less than 25%, there is no study heterogeneity; if the value of *I^2^* is between 50% and 74%, there is moderate heterogeneity; and if the value of *I^2^* is 75% or more, there is high heterogeneity. Egger’s test was used to assess study bias.

## Results

### Search results and characteristics of included studies

**Figure 1.**
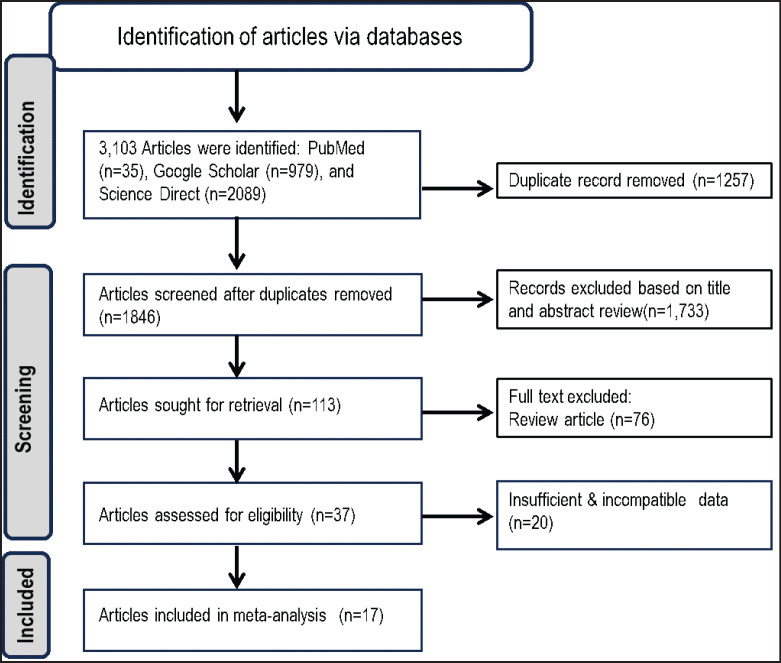
The PRISMA flow chart describes the number of articles identified, screened, and included for eligibility in this study (Adapted from [49]).

In the literature review, 3,103 articles were initially retrieved from electronic databases: PubMed (*n* = 35), Google Scholar (*n* = 979), and ScienceDirect (*n* = 2,089). Duplicate articles (*n* = 1,257) were excluded. The remaining 1,846 articles were title and abstract reviewed, and 1,733 were excluded. Furthermore, out of the remaining 113 articles, a full-text review was performed; 76 review articles and 20 articles with incomplete or inconsistent information were excluded (Fig. 1). Finally, 17 articles met the inclusion criteria for this meta-analysis [28–32, 38–48]. The detailed features of the included studies, such as study location, design, size, pro-oxidant and antioxidant markers, and NOS score, are shown in Table 1.

### Meta-analysis results of pro-oxidant markers

**Figure 2.**
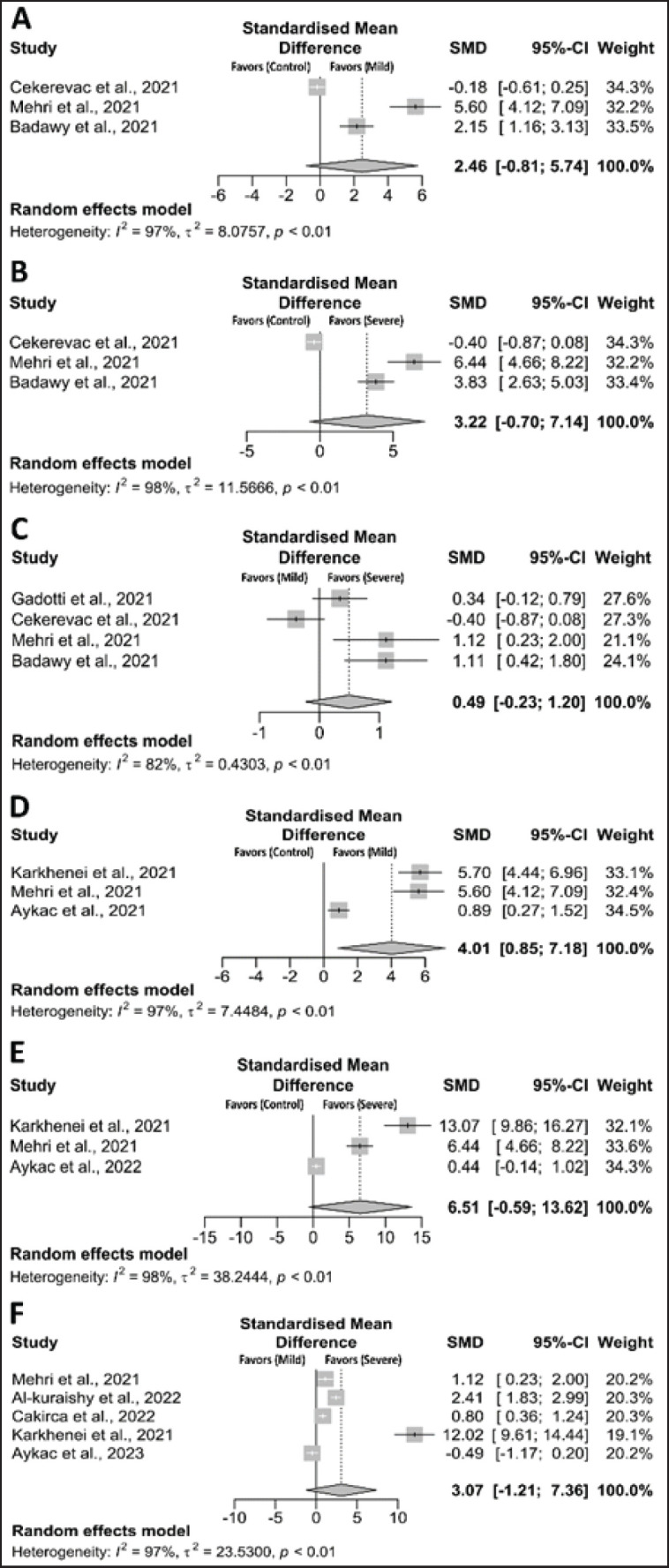
Forest plot of the pro–oxidant markers between COVID-19 (mild or severe) and healthy individuals. (A) H₂O₂ (control *vs.* mild), (B) H₂O₂ (control *vs.* severe), (C) H₂O₂ (mild *vs.* severe), (D) TOS (control *vs.* mild), (E) TOS (control *vs.* severe), and (F) TOS (mild *vs.* severe).

Compared to controls versus mild cases, controls versus severe cases, and mild versus severe cases, H₂O₂ levels were found to be significantly higher [(SMD = 2.46; CI: –0.81 to 5.73; *p* = 0.05, *I^2^* = 97%), (SMD = 3.22; CI: –0.70 to 7.14; *p* = 0.05, *I^2^* = 98%), and (SMD = 0.49; CI: –0.23 to 1.20; *p* = 0.05, *I^2^* = 82%), respectively] (Fig. 2A–C). Similarly, TOS levels were found to be significantly higher when compared to controls versus mild cases (SMD = 4.01; CI: 0.85 to 7.18; *p* = 0.013, *I^2^* = 97%), controls versus severe cases (SMD = 6.51; CI: –0.59 to 13.62; *p* = 0.07, *I^2^* = 98%), and mild versus severe cases (SMD 3.07; CI: 01.21 to 7.36; *p* = 0.05, *I*^2^ = *I^2^* = 97%) (Fig. 2D–F). The adjusted SMDs from the trim and fill method also showed significant changes in H₂O₂ and TOS between control and mild, control and severe, and mild and severe patients (Table 2).

**Table 1.** Summary information of the included studies.

Sl No.	Reference	Study location	Sample size (*n*)			Pro-oxidant/antioxidant markers	Study design	NOS score
			Control	Mild	Severe			
1	Mehri et al. [30]	Hamadan (Iran)	24	14	10	H_2_O_2_, CAT, TOS	Case-control	8
2	Montiel et al. [31]	Brussels (Belgium)	15	30	30	NO	Observational	7
3	Badawy et al. [29]	Egypt	11	16	23	H_2_O_2_	Observational	8
4	Yaghoubi et al. [32]	Mashhad (Iran)	60	60	60	SOD, NO, CAT	Cross-sectional comparative	7
5	Žarković et al. [38]	Zagreb (Croatia)	34	66	22	SOD	Case–control	7
6	Cekerevac et al. [28]	Kragujevac (Serbia)	35	48	33	H_2_O_2_, SOD, NO	Observational and cross-sectional	8
7	Al-Kuraishy et al. [40]	Baghdad (Iraq)	–	39	41	TOS	Single-center cohort	8
8	Çakırca et al. [41]	Sanliurfa (Turkey)	–	46	40	TOS, TT	Prospective, single-center	7
9	Kalem et al. [42]	Turkey (Ankara)	70	117	27	TT	Prospective cohort	8
10	van Eijk et al. [44]	Groningen (Netherland)	30	29	29	TT	Prospective cohort	7
11	Erel et al. [23]	Turkey (Ankara)	70	90	82	TT	Case-control	8
12	Karkhenei et al. [50]	Iran	18	35	19	TOS, GSH	Case-control	8
13	Gadotti et al. [33]	Brazil	–	44	33	H_2_O_2_	Prospective cohort	8
14	Aykac et al. [45]	Turkey	34	16	18	TOS, TT	Prospective cohort	8
15	Coronel et al. [46]	Brazil	20	–	15	CAT	Observational	7
16	Neves et al. [47]	Brazil	–	95	20	GSH	Prospective cohort	8
17	Mete et al. [48]	Turkey	43	–	43	TT	Observational	8
	Total		464	745	545			

CAT, Catalase; GSH, glutathione; H_2_O_2_, Hydrogen peroxide; NO, nitric oxide; SOD, superoxide dismutase; TOS, total oxidative stress; TT, total thiols;.

### Meta-analysis results of antioxidant markers

**Figure 3.**
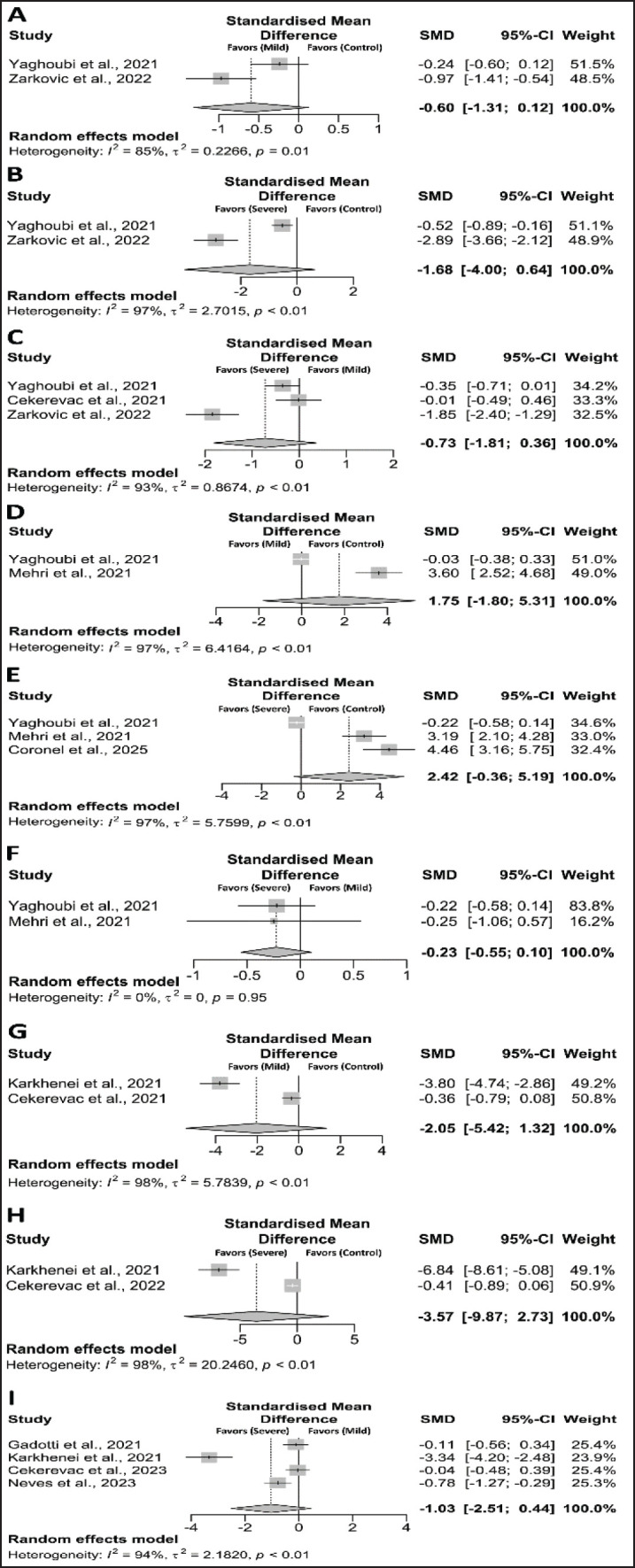
Forest plot of the antioxidant markers between diseased and control/healthy individuals. (A) SOD (control *vs.* mild), (B) SOD (control *vs.* severe), (C) SOD (mild *vs.* severe), (D) CAT (control *vs.* mild), (E) CAT (control *vs. *severe), (F) CAT (mild *vs.* severe), and (G) GSH (mild *vs.* severe).

**Figure 4.**
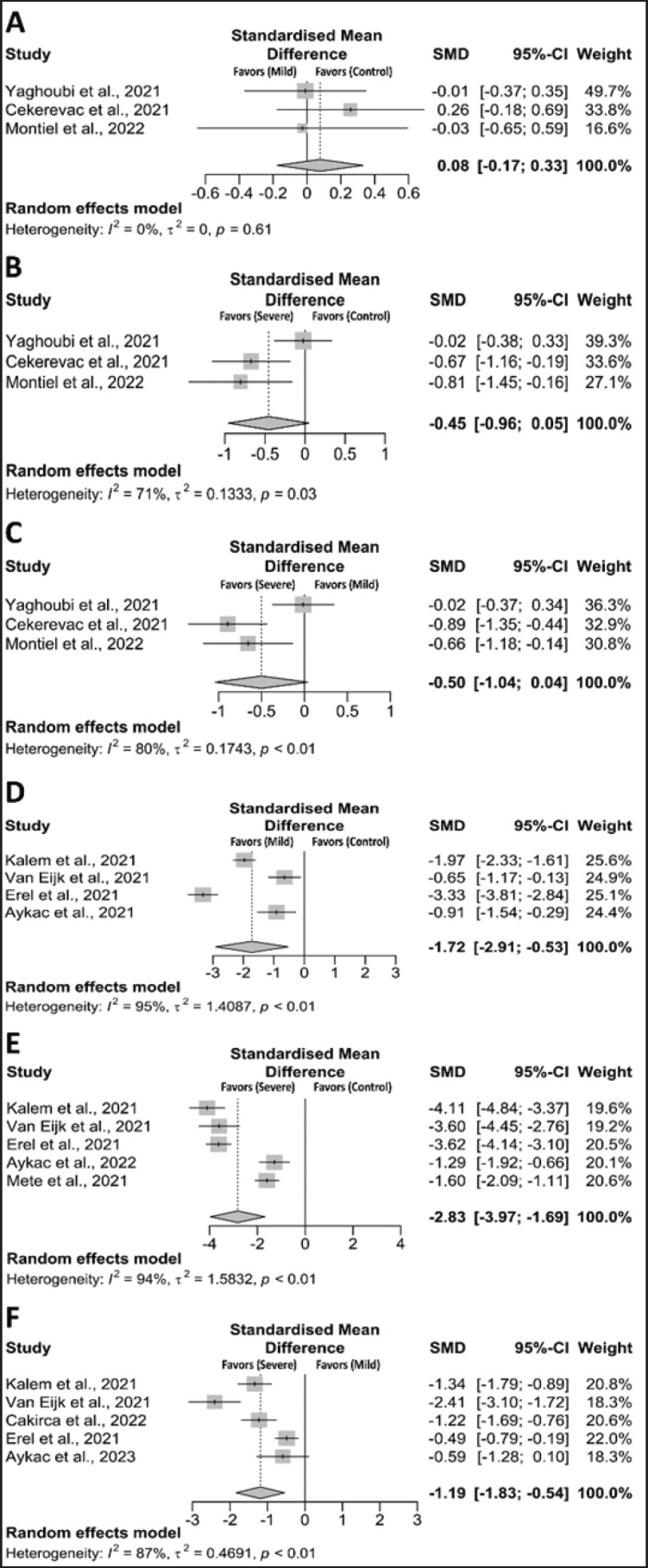
Forest plot of the antioxidant markers between COVID–19 and healthy individuals. (A) NO (control *vs*. mild), (B) NO (control *vs*. severe), (C) NO (mild *vs*. severe), (D) thiol (control *vs*. mild), (E) thiol (control *vs*. severe), and (F) thiol (mild *vs*. severe).

When we compared control vs mild cases, control vs severe cases, and mild vs severe cases, the levels of SOD were found to be significantly lower [(SMD, –0.60; CI: –1.31 to 0.12; *p* = 0.05), (SMD, –1.68; CI: –4.00 to 0.64; *p* = 0.05), and (SMD, –0.73; CI: –1.81 to 0.36; *p* = 0.06), respectively]. (Fig. 3A–C). Similarly, CAT levels were found to decrease when comparing control versus mild cases (SMD, 1.75; CI: –1.80 to 5.31; *p* = 0.334), control versus severe cases (SMD, 2.42; CI: –0.36 to 5.19; *p* = 0.057), and mild versus severe cases (SMD, –0.23; CI: –0.55 to 0.10; *p* = 0.056) (Fig. 3D–F). GSH levels were found to decrease when comparing control versus mild cases (SMD, –2.05; CI: –5.42 to 1.32; *p* = 0.234), control versus severe cases (SMD, –3.57; CI: –9.87 to 2.73; *p* = 0.267), and mild versus severe cases (SMD, –1.03; CI: –2.51 to 0.44; *p* = 0.170) (Fig. 3G–I). Moreover, thiol levels were significantly decreased when comparing control versus mild cases (SMD, –1.72; CI: –2.91 to –0.53; *p* = 0.005), control versus severe cases (SMD, –2.83; CI: –3.97 to –1.69; *p* = 0.001), and mild versus severe cases (SMD, –1.19; CI: –1.83 to –0.54; *p* = 0.001) (Fig. 4D–F). In addition, nitric oxide (NO) levels were significantly decreased when comparing control versus mild cases (SMD, 0.08; CI: –0.17 to 0.33; *p* = 0.546), control versus severe cases (SMD, –0.45; CI: –0.96 to 0.05; *p* = 0.058), and mild versus severe cases (SMD, –0.50; CI: –1.04 to 0.04; *p* = 0.057) (Fig. 4A–C). Similarly, the adjusted SMD from the trim and fill method also showed significant changes in thiol and NO between control and mild, control and severe, and mild and severe patients (Table 2).

### Publication bias

We assessed the publication bias of the literature using Egger’s test for all studies included in each index (Table 2). Funnel plots are a widely used technique for detecting publication bias. However, this requires at least 10 studies [51]. To achieve the same objective, we used Egger’s test and obtained *p*-values greater than 0.05 (Table 2). If the *p*-value is less than 0.05, publication bias is indicated [51]. Thus, these results indicate that our publications were unbiased.

**Table 2.** Summary information on the pro-oxidant and antioxidant markers results from the forest plots and the trim and fill method.

Markers	Comparison between	Studies	Egger’s test (*p* =)	SMD	95% CI	*p*-value (SMD)	Unbiased results according to the trim and fill method		
							SMD	95% CI	*p* =
H_2_O_2_	Control *vs.* mild	3	0.087	2.46	–0.81 to 5.73	0.054	–0.17	–4.00 to 3.65	0.052
	Control *vs.* severe	3	0.036	3.22	–0.70 to 7.14	0.051	–0.39	–5.34 to 4.55	0.058
	Mild *vs.* severe	4	0.131	0.49	–0.23 to 1.20	0.058	0.06	–0.66 to 0.80	0.065
TOS	Control *vs.* mild	3	0.119	4.01	0.85 to 7.18	0.013	0.89	–3.26 to 5.05	0.057
	Control *vs.* severe	3	0.012	6.51	–0.59 to 13.62	0.072	0.44	–8.14 to 9.02	0.059
	Mild *vs.* severe	5	0.232	3.07	–1.21 to 7.36	0.0.05	3.07	–1.21 to 7.36	0.051
SOD	Control *vs.* mild	2		–0.60	–1.31 to 0.12	0.051			
	Control *vs.* severe	2		–1.68	–4.00 to 0.64	0.051			
	Mild *vs.* severe	3	0.559	–0.73	–1.81 to 0.36	0.061	–0.72	–1.81 to 0.36	0.071
CAT	Control *vs.* mild	2		1.75	–1.80 to 5.31	0.334			
	Control *vs.* severe	3	0.025	2.42	–0.36 to 5.19	0.057	–0.22	–3.79 to 3.35	0.052
	Mild *vs.* severe	2		–0.23	–0.55 to 0.10	0.056			
GSH	Control *vs.* mild	2		–2.05	–5.42 to 1.32	0.234			
	Control *vs.* severe	2		–3.57	–9.87 to 2.73	0.267			
	Mild *vs.* severe	4	0.012	–1.03	–2.51 to 0.44	0.170	–1.03	–2.51 to 0.44	0.170
NO	Control *vs.* mild	3	0.996	0.08	–0.17 to 0.33	0.546	0.07	–0.17 to 0.33	0.546
	Control *vs.* severe	3	0.245	–0.45	–0.96 to 0.05	0.056	–0.02	–0.63 to 0.59	0.040
	Mild *vs.* severe	3	0.356	–0.50	–1.04 to 0.04	0.057	–0.01	–0.68 to 0.65	0.062
Thiol	Control *vs.* mild	4	0.631	–1.72	–2.91 to –0.53	0.005	–1.72	–2.91 to –0.53	0.005
	Control *vs.* severe	4	0.466	–2.83	–3.97 to –1.69	0.0001	–2.82	–3.97 to –1.69	0.0001
	Mild *vs.* severe	5	0.214	–1.19	–1.83 to –0.54	0.0001	–1.18	–1.83 to –0.54	0.0001

CAT, Catalase; H_2_O_2_, Hydrogen peroxide; GSH, glutathione; NO, nitric oxide; SOD, superoxide dismutase; TOS, total oxidative stress; TT, total thiols. Forest plots and the trim and fill method under a random–effects model based on the standardized mean difference (SMD). Egger’s test was used to assess study bias.

### Sensitivity analysis

Significant heterogeneity was detected across all comparisons (Figs. 2–4). Sensitivity analysis showed that excluding any specific studies for H₂O₂, TOS, SOD, CAT, GSH, thiols, and NO within the control to mild cases, control to severe cases, and mild to severe cases did not affect our results. Sensitivity analysis showed that excluding any specific studies for H₂O₂, TOS, SOD, CAT, GSH, thiols, and NO between the control-to-mild, the control-to-severe, and the mild-to-severe did not affect our results (data not shown), suggesting that it is better to keep this result in the meta-analysis. Thus, our sensitivity analysis indicates that most of our results are reliable.

## Discussions

This meta-analysis explored the connection between oxidative stress and the severity of COVID-19. First, we examined pro-oxidant markers, H₂O₂, and TOS, and found they were significantly elevated in both mild and severe patients compared to healthy individuals. Next, we assessed levels of intracellular antioxidant markers, SOD and CAT, and observed that they were significantly reduced in mild and severe cases relative to healthy controls. Finally, we measured thiol and its derivative, GSH levels, along with NO, and found notable decreases in mild and severe COVID-19 patients compared to controls. These findings align with a hospital cohort study that reported pro-oxidant and antioxidant gene polymorphisms are linked to COVID-19 severity [52].

Accumulating evidence indicates that the pathophysiological underpinnings of COVID-19 severity are associated with oxidative stress [14]. On the other hand, NAC and vitamin C, antioxidant therapies, have shown positive outcomes for variant-independent SARS-CoV-2 infection [25, 53]. Although meta-analysis on pro-oxidant and antioxidant markers in monitoring COVID-19 severity has not yet been conducted, the current study, which includes 17 papers with 1,754 participants, found that oxidative stress is associated with COVID-19 severity. Several clinical cohort studies have found similar findings. For example, a clinical cohort study in Serbia found that O₂•− and •OH levels were significantly higher in severe COVID-19 patients compared to mild to moderate patients [28]. In another study, more than 80% of severe COVID-19 patients had a neutrophil-lymphocyte ratio (NLR) value greater than 5, and more than 80% of non-severe patients had a value less than 5 [54]. Indeed, high levels of NLR increased free radical production, leading to a redox imbalance that drives the pathophysiology of COVID-19 severity [12, 15]. Furthermore, neutrophil-associated pro-oxidant markers, such as H₂O₂ and O₂•−, were twice as high in survivor COVID-19 patients and three times higher in non-survivor patients than in controls [29]. Similarly, significantly higher TOS levels were observed in ICU patients than in non-ICU patients [41]. An observational study of 152 individuals in a Mexican hospital showed that COVID-19 patients (*n* = 76) had higher levels of malondialdehyde (MDA), a potent oxidative stress marker, and lower total antioxidant capacity (TAC) levels compared to healthy controls (*n* = 76) [55]. These suggest that increased production of pro-oxidants significantly contributes to COVID-19 pathogenesis.

The current study also examined the levels of antioxidant markers like SOD and CAT and found them to be significantly decreased in severe COVID-19 patients. These findings are consistent with several clinical cohort studies. With one, excessive pro-oxidant production and dysfunction of the antioxidant system were associated with the severity of COVID-19 [30]. Another study conducted in an Iranian hospital showed that TAC, SOD, CAT, and NO levels were significantly reduced in severe COVID-19 patients (*n* = 120) compared to healthy individuals (*n* = 60) [32]. Similarly, serum levels of SOD and CAT were found to be significantly lower in non-survivors compared to COVID-19 survivors [47]. In addition, immunohistochemical analysis of lung autopsy results showed that SOD levels were decreased in both pneumocytes and alveolar macrophages of individuals who died of COVID-19 compared to healthy individuals [56]. Furthermore, a case-control study conducted at Persian Gulf Shahid Hospital, Bushehr University of Medical Sciences, Iran, from May 2021 to September 2021 showed that SOD and NO levels were significantly decreased and MDA levels were increased in severe patients (*n* = 300) compared to the mild (*n* = 300) and normal groups (*n* = 150) [57]. A recent study has shown high levels of oxidative damage in severe and critically ill COVID-19 patients, indicating a hallmark of the severity of COVID-19 patients [58].

GSH is another potent antioxidant that reduces viral load and infectivity, inhibits oxidative stress, pro-inflammatory cytokine release, and thrombosis production, and potentially enhances immune function. Conversely, reduced levels of GSH are a potential factor in susceptibility to SARS-CoV-2 infection [59]. In this meta-analysis, thiols and GSH levels were significantly reduced in severe COVID-19 patients compared to healthy individuals, and significant reductions were also found in mild and severe COVID-19 patients. This finding is supported by a clinical cohort study in which severe COVID-19 patients had severe GSH deficiency, increased oxidative stress, and higher oxidative damage compared to controls [43]. In another hospital-based observational study, 587 subjects (517 patients/70 healthy) showed that a graded decrease in thiol levels was closely associated with the progression of severe COVID-19 [23]. Furthermore, GSH and thiol levels were significantly reduced in 78 hospitalized patients with COVID-19 compared to healthy controls [50]. Similarly, a study of 115 patients at a public hospital in Brazil found that a decrease in serum GSH levels below 327.2 μmol/ml was associated with a significant risk of death in COVID-19 patients [47]. Conversely, our previous meta-analysis found that NAC supplementation improved clinical outcomes in COVID-19 patients [25]. Indeed, NAC, the precursor of GSH, acts as a potent antioxidant by scavenging ROS by interacting with a free thiol [60]. GSH participates in electron-donating redox reactions that detoxify ROS [61].

A clinical trial of vitamin C showed potential benefits in improving oxygenation in critically ill COVID-19 patients [62]. Furthermore, vitamin C has been shown to increase the production of interferons, which enhances antiviral responses [63]. Moreover, vitamin C has been shown to reduce inflammation, even cytokine storms, by inhibiting the nuclear factor kappa B pathway [64]. Thus, vitamin C reduces oxidative stress, thereby improving endothelial cell integrity and wound healing, a potentially beneficial strategy for preventing early and severe SARS-CoV-2 infection.

**Figure 5.**
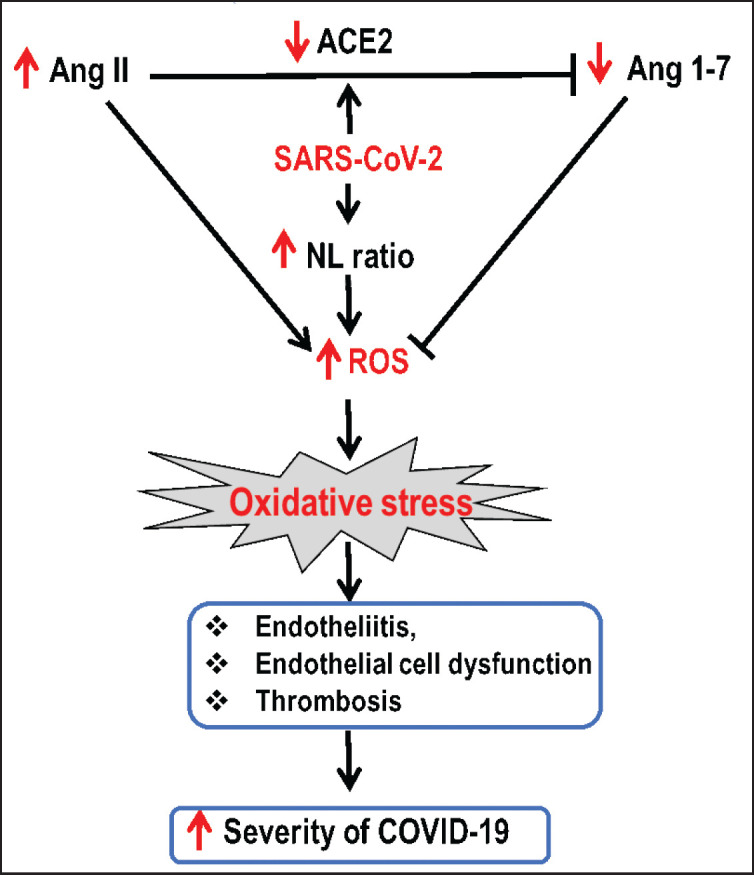
Potential mechanisms of oxidative stress and COVID–19 severity caused by SARS-CoV-2. SARS-CoV-2 blocks ACE–2 from converting Ang II to Ang 1–7, promoting ROS generation. Moreover, SARS-CoV-2 infection directly increases the production of ROS by increasing the NLR via activation of the NADPH oxidase pathway. The oxidative stress causes endothelial cell dysfunction that activates the blood coagulation cascade, leading to thrombosis associated with disease severity.

This meta-analysis revealed a noteworthy finding that oxidative stress is associated with the severity of COVID-19. Although clinical data on the molecular mechanisms underlying increased ROS production during SARS-CoV-2 infection are limited, we will describe several general pathways of ROS accumulation that may contribute significantly to the severity and high mortality of COVID-19 (Fig. 5). First, the generation of free radicals and oxidative stress in COVID-19 patients are related to ACE–2. Physiologically, ACE–2 converts angiotensin (Ang) II to Ang 1–7 [65]. In fact, Ang II is a potent stimulator of nicotinamide adenine dinucleotide phosphate (NADPH) oxidase that promotes O₂•− and H₂O₂ production; conversely, Ang 1–7 inhibits O₂•− and H₂O₂ production, thereby maintaining oxidant-antioxidant homeostasis in the body [66]. During SARS-CoV-2 infection, the availability of “free” ACE–2 is reduced due to virus binding and/or entry into cells, leading to increased Ang II and decreased Ang 1–7 levels, which stimulate NADPH oxidase activity and increase ROS production [67–69]. Second, SARS-CoV-2 infection directly increases ROS production by increasing NLRs. Higher NLR was found in non-survivor patients than in mild/survivors [70, 71]. In fact, in COVID-19 infection, neutrophils and macrophages cause excessive ROS production (respiratory burst) while destroying pathogens (phagocytic components) via NADPH oxidase [70–74]. Third, decreased GSH levels in hospitalized COVID-19 patients are associated with increased oxidative stress [50]. In contrast, optimal levels of GSH are crucial for the functioning of the innate immune system and the reduction of oxidative stress [22]. Fourth, mitochondrial dysfunction leads to an increase in ROS production. COVID-19 has been shown to disrupt mitochondria by producing toxic gases, such as hydrogen sulfide [75]. Furthermore, H₂O₂ activates pro-inflammatory cytokines in macrophages, neutrophils, and endothelial cells, generating more O₂•− and H₂O₂ through NADPH oxidase [73, 75].

Although COVID-19 is generally considered a respiratory disease, blood vessels are the primary target when developing into severe disease [14, 76]. The pathophysiological feature of COVID-19, oxidative stress, affects blood vessels by altering immune cell function and hyperinflammatory response [77]. This results in endothelial cell dysfunction that activates the blood clotting cascade, which subsequently causes vascular thrombosis [14]. The thrombus can break up into small emboli and then flow into small blood vessels, where they can become trapped and cause ischemia and tissue necrosis (Fig. 5).

The highest rates of severe COVID-19 illness and mortality are found in patients who are typically over 60 years of age and have underlying comorbidities such as hypertension, diabetes, cancer, obesity, and immunosuppressive conditions [13, 14]. Although clinical data on the antioxidant system in elderly and comorbid patients during SARS-CoV-2 infection are limited, the increased basal levels of pro-oxidants and decreased levels of antioxidants in metabolic diseases with aging have led to the idea that oxidative stress may contribute significantly to COVID-19 severity and higher mortality [12, 15, 73]. For example, decreased levels of SOD have been observed in the lungs of elderly patients with COVID-19 and have been suggested to contribute to increased disease severity [78]. Lower GSH levels have been observed in older individuals and COVID-19 patients with comorbidities associated with severe illness and death [59]. In a cross-sectional comparative study, endogenous (SOD, CAT, and GPx) and exogenous antioxidants (vitamins A, C, and E, and Se, Zn, Mg, and Cu) were significantly reduced in patients infected with SARS-CoV-2 compared with healthy individuals [79]. Increased levels of NADPH oxidase-induced oxidative stress were observed in patients with underlying comorbidities, suggesting that oxidative stress plays an important role in the progression of COVID-19 severity and mortality, especially in the elderly and those with comorbidities.

To the best of our knowledge, this is the first meta-analysis on the association of oxidative stress with the severity of COVID-19. Strengths of this study include comprehensive systematic search strategies, data abstraction, and a predefined protocol for a comprehensive quality assessment of primary research. We used internationally recognized critical appraisal tools to assess the quality of individual studies. Of course, this study also has some limitations. First, although we systematically searched the literature to identify eligible studies, some studies might have been missed. Despite the extensive search strategy, the databases we searched did not index non-English language studies. Second, this meta-analysis included 17 articles involving 1,754 participants (464 controls/745 mild/545 severe COVID-19), which is a small number to predict the relationship of oxidative stress with COVID-19 severity. Third, this meta-analysis contains a mixture of case-control, observational, prospective, and cohort studies and may have some concerns about the risk of heterogeneity. Fourth, because the number of studies in each group was less than 10, we could not conduct a funnel-plot analysis to determine study bias; therefore, NOS and Egger’s tests were conducted. Egger’s test *p* > 0.05 for all considered groups, indicating unbiased despite a high percentage of heterogeneity. Finally, some studies did not distinguish the comorbidities in the elevation of these markers; therefore, it is difficult to conclude whether the severity of COVID-19 is due to oxidative stress. Despite some limitations, our study provides an important foundation for a broader understanding of COVID-19 severity and oxidative stress.

## Conclusion

Our synthesized results revealed significantly higher levels of pro-oxidants (H₂O₂ and TOS) and lower levels of antioxidants (SOD, CAT, GSH, thiols, and NO) in severe cases of COVID-19 compared to controls and mild cases. This information will be valuable for a broader discussion on the pathogenesis of COVID-19. Since the number of included studies was small, large-scale clinical studies are needed to explore the role of oxidative stress in severe COVID-19. A better understanding and regular monitoring of oxidative stress may pave the way for future efforts to reduce COVID-19-induced complications and severity.

## List of abbreviations

ACE2, angiotensin-converting enzyme 2; CAT, catalase; COVID-19, coronavirus disease 2019; GSH, glutathione; H₂O₂, hydrogen peroxide; ICU, intensive care unit; MDA, malondialdehyde; NAC, N–acetylcysteine; NAC, N–acetylcysteine; NADPH oxidase, nicotinamide dinucleotide phosphate oxidase; NL ratios, neutrophil–lymphocyte ratios; O₂•−, superoxide; PRISMA, preferred reporting items for systematic reviews and meta-analysis; ROS, reactive oxygen species; SARS-CoV-2, Severe acute respiratory syndrome coronavirus-2; SOD, superoxide dismutase; TAC, total antioxidant capacity; TOS, total oxidative stress; TT, total thiol.
